# Investigating protein C and S levels in pregnant women with recurrent early pregnancy loss *versus* normal pregnancy

**DOI:** 10.25122/jml-2022-0267

**Published:** 2023-01

**Authors:** Beenish Mukhtar, Rinku Garg, Guru Ibrahim, Jyoti Batra

**Affiliations:** 1 Department of Physiology, Santosh Deemed to be University, Ghaziabad, India; 2 Department of Physiology, College of Medicine, Dar Al Uloom University, Riyadh, Kingdom of Saudi Arabia; 3 Department of Physiology, School of Medical Sciences and Research, Sharda University, Greater Noida, India; 4 Department of Gynaecology and Obstetrics, Guru Multi Speciality Hospital, Kashmir, India; 5 Department of Biochemistry, Santosh Deemed to be University, Ghaziabad, India

**Keywords:** deficiency, protein C, protein S, pregnancy loss

## Abstract

Miscarriage in the first and second trimesters of pregnancy is very common, and coagulopathy can be a contributing factor. Protein C and S deficiency are rare, inherited disorders that can increase the risk of thrombophilia. Women with these deficiencies have a higher risk of developing blood clots in the placenta, which can lead to placental insufficiency and, ultimately, to a miscarriage. We aimed to compare the levels of protein C and protein S in pregnant females with recurrent first and second-trimester pregnancy loss and normal pregnant females. We performed a detailed history, examination, and various lab tests on a cohort of 40 females with a history of recurrent first and second-trimester abortions visiting an outpatient clinic at a multi-specialty hospital in Kashmir, India. All the findings were compared with 40 women with normal pregnancies. 10% of the participants had low protein C and S levels (P=0.277), out of whom 75% (p<0.001) had intrauterine growth retardation (IUGR) on ultrasound with 67% (p<0.001) having reduced doppler flow in the umbilical artery. 0.05% of participants had isolated protein S deficiency with no concomitant IUGR seen. Patients with protein C and S deficiencies were treated with heparin and progesterone and followed up for pregnancy outcomes. Screening for protein C and S deficiency is mandatory in all cases of recurrent pregnancy loss. Treatment with low molecular weight heparin and progesterone should be initiated to ensure good fetal outcomes and prevent post-partum/postoperative catastrophic venous thromboembolism events.

## INTRODUCTION

Proteins C and S are vitamin K-dependent plasma proteins that work together as an integral part of the body’s natural anticoagulant system. They work through the selective inactivation of Factors Va and VIIIa. Mutations in protein C and S genes are the main causative factors for their deficiencies. In addition, acquired deficiencies can also occur due to multiple mechanisms and/or the use of certain drugs [1]. This condition demands even more attention among pregnant women, given the hypercoagulable state of pregnancy. Normal pregnancy is associated with increased procoagulants, decreased fibrinolysis, and decreased anticoagulants to maintain placental hemostasis during pregnancy [2]. However, hypercoagulability due to deficiency of any antithrombotic factors leads to placental thrombosis, hypoperfusion, fetal growth retardation, fetal death, and subsequent fetal losses. Functional protein C or S levels do not typically change significantly during a normal pregnancy. Only the levels of free protein S fall significantly during the first and second trimesters of pregnancy, but there is no further decrease during the third trimester. Protein S deficiency is more commonly identified than protein C deficiency, constituting an overall 15-fold increased risk of recurrent pregnancy loss. Therefore, thrombophilia screening in women who have experienced pregnancy loss may be appropriate, and treatment with low molecular weight heparin (LMWH) may be an option [3, 4]. Our study aimed to: (1) estimate the levels and prevalence of serum proteins C and S in pregnant women with a history of recurrent miscarriages and (2) compare these with the levels of pregnant women with no history of miscarriage.

## MATERIAL AND METHODS

This study was conducted at Santosh Medical College, Department of Physiology, in collaboration with the Department of Obstetrics and Gynaecology, Guru Multi Speciality Hospital, Kashmir. Ethical clearance was obtained from the institutions before carrying out the study.

This prospective cross-sectional study was conducted over one year, from Dec 2020 to Dec 2021. Given the low prevalence of protein C and S deficiency worldwide, which is estimated to be around 3 cases per thousand individuals, the presumed prevalence value of 0.003 was used in the PASS software while applying the Exact Clopper Pearson method. The estimated sample size for the prospective study group was determined to be n=38. This sample size provides a two-sided 95% confidence interval with a width of 0.098.

Using a mathematical relationship between the F distribution and the cumulative binomial distribution, the lower and upper confidence limits of a 100 (1-a)% exact confidence interval for the true proportion p are given by:


$$p=\hat{p}\pm Z_{\left(1-\alpha /2\right)}\sqrt{\frac{\hat{p}(1-\hat{p})}{n}}$$

In the present study, 80 pregnant women were recruited and divided into two groups for analysis. Group A, also known as the case group, consisted of 40 women who experienced three or more miscarriages in the first or second trimester of pregnancy. Group B, also known as the control group, consisted of 40 healthy pregnant women in their third trimester.

We collected a detailed history from each participant, including demographic profile and comprehensive obstetric and drug history. Additionally, we collected data on their current symptoms, including leg pain, chest discomfort with breathlessness, weakness on one side of the body, and history of deep vein thrombosis (DVT), stroke, low birth weight (LBW), and intrauterine growth retardation (IUGR). Following this, a detailed general and local examination was performed by the concerned gynecologist. This examination included checking vital signs, body mass index (BMI) and performing an abdominal and pelvic examination. Finally, a series of routine laboratory tests were recommended (complete blood count, urine analysis, blood glucose, thyroid profile, Protein C and S), and an ultrasound was performed. Hypertensive patients received multivitamins, progesterone support, and amlodipine.

In addition to the laboratory tests, we also analyzed the antenatal ultrasound for each case. Most of the cases showed normal results on ultrasound, except for a few cases with low levels of protein C and S, which showed signs of IUGR on ultrasound. To further investigate these cases, we performed a Doppler flow study to evaluate any signs of reduced umbilical blood flow, which could indicate the presence of thrombosis. Patients were followed up with laboratory results, and those with low protein C and S levels were prescribed low molecular weight heparin (LMWH) by the gynecologist as an additional treatment to prevent thrombotic events.

### Determining the levels of proteins C and S

A 5 ml venous sample was drawn from the patient under all aseptic precautions. The samples were collected into siliconized glass tubes containing 0.1 mol/l buffered sodium citrate. Platelet-poor plasma was prepared by centrifugation at 2000 g for 15min at room temperature. The samples were loaded into the fully automated coagulation ASEKULISA analyzer. The AESKULISA Protein C and S is a sandwich ELISA using microplates coated with a capture antibody specific for human protein C or S. 1:51 diluted patient plasma is incubated in the wells allowing protein C and S present in the plasma to bind to the antibody. The unbound fraction is removed by washing. Afterward, the anti-human protein C or S detection antibody conjugated to horseradish peroxidase (conjugate) is incubated and reacts with the antigen-antibody complex on the microwell surface. Following incubation, the unbound conjugate is washed off. Adding TMB-substrate generates an enzymatic colorimetric (blue) reaction, which is stopped by diluted acid (color changes to yellow). The rate of color formation from the chromogen is measured in optical density units with a spectrophotometer at 450 nm. Using a curve prepared from the Reference Plasma provided with the kit, the Protein C and S antigen relative percent concentration in patient plasma can be determined.

We analyzed and compared the results between cases and controls. For patients with low protein C and S levels, we looked for correlations between lab results and other factors such as medical history, family history, blood pressure, ultrasound findings, and treatment. Furthermore, we also compared patients with combined deficiency of both proteins to those with isolated deficiency of one. Patients with reduced levels of protein C and S were followed-up to monitor the pregnancy outcome.

Inclusion criteria: pregnant females between 20 to 50 years during the first and second trimesters with a history of recurrent pregnancy loss, visiting the department of Gynae and Obstetrics at the Guru Multi Speciality hospital. The inclusion criteria for the control group were women in their third trimester of pregnancy.

Exclusion criteria: women in their third trimester (for the case groups) or with other causes of pregnancy loss:

- Genital tract malformations;
- Fetal malformations;
- Infectious causes [*e.g*., sexually transmitted infections or TORCH infections (toxoplasma, other infections, rubella, cytomegalovirus, herpes simplex)];
- ABO and Rh incompatibility;
- Diabetes mellitus.

### Statistical analysis

The statistical analysis was carried out using SPSS version 20. Categorical variables were presented as frequencies and percentages. Pearson’s chi-square (X^2^) test and Fisher exact test were used to assess the relationship between categorical variables. A p-value of ≤0.05 was considered significant.

## RESULTS

### Demographic characteristics of participants

**Figure 1:**
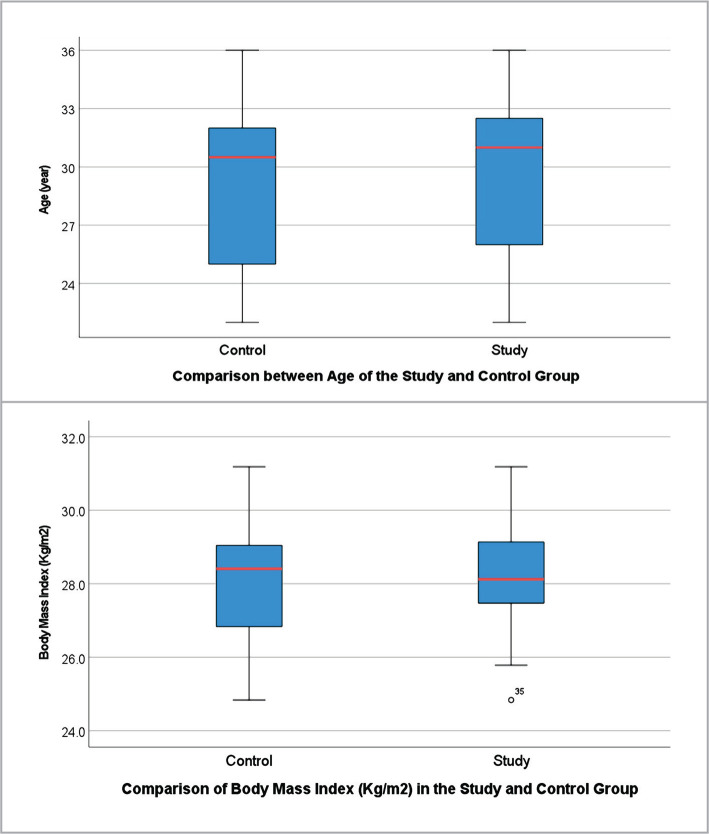
Demographic profile of study and control groups.

The mean age of females in the study group was 29.8±4.1, and in the control group, 29.4±4.3. The mean BMI of females in the study group was 28.1±1.4, and 28.1±1.9 in the control group (Figure 1).

### Ultrasound results among groups

**Figure 2:**
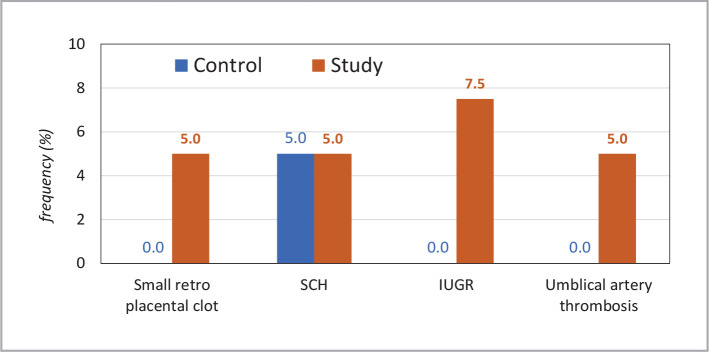
Ultrasound findings in the study and control groups.

Regarding ultrasound findings, 33 cases had normal results throughout their pregnancy, 2 had a small retroplacental clot, and 2 had a subchorionic hemorrhage. Three females in the case group had IUGR, and 2 of them showed reduced umbilical artery flow on Doppler, indicating suspected thrombosis. In the control group, 38 patients had normal ultrasound results, and 2 had a subchorionic hemorrhage. There were no reported cases of IUGR or umbilical artery abnormalities in the control group (Figure 2).

### The mean levels of protein C and S in the study and control groups

Nine cases were positive for antinuclear antibodies (ANA), compared to only 1 in the control group. 10 cases showed anemia in their complete blood count (CBC), while 8 of the controls were anemic. Protein C and S levels were significantly decreased in 4 out of 40 cases, but none of the controls had decreased levels of either protein. Additionally, we observed lower levels of isolated free protein S in 2 of the cases (Table 1).

**Table 1:** Protein C and S in study and control groups (Mean±SD).

Characteristic	Description	Control	Study	p-value
**ANA**	+Ve	1 (2.5)	9 (22.5)	0.014
**CBC**	Anemia	8 (20.0)	10 (25.0)	0.79
**Protein C level**	min–max	85–134	50–134	0.277
	Median (P25–P75)	103.5 (96.5–10)	103 (93–110)	
	Mean±SD	104.5±12.2	100.6±18.8	
	Low (<70)	0 (0.0)	4 (10.0)	
**Protein S level (free)**	min–max	73–124	41–124	0.058
	Median (P25–P75)	89.5 (79–97)	90 (78–97)	
	Mean±SD	90.8±13.9	83.6±19.1	
	Low (<60)	0 (0.0)	6 (15.0)	

### Parameters across combined protein C and S deficiency

Out of the 4 cases with low protein C and S levels, only one had normal ultrasound results, while 3 had IUGR, and 2 showed low umbilical artery flow, indicating suspected thrombosis. Three of these cases had a history of IUGR, and 4 had a history of abortions. All 4 cases had a positive family history of abortions, and none had a history of deep vein thrombosis. All 4 of them were treated with LMWH and progesterone support. Two cases were hypertensive and were treated with Amlong (Table 2).

**Table 2:** Parameters across combined protein C and S deficiency.

		Normal (70–140)	Low (<70)	P-value
**SBP**	min–max	100–134	126–145	0.008
	Mean±SD	117.6±9.8	135.3±8.8	
**DBP**	min–max	60–90	70–97	0.528
	Mean±SD	73.7±7.8	79.3±12.7	
**USG findings for present pregnancy**	WNL	32 (97.0)	1 (3.0)	0.013
	Small retro placental clot	2 (100.0)	0 (.0)	1.000
	SCH	2 (100.0)	0 (.0)	1.000
	IUGR	0 (.0)	3 (75.0)	<0.001
	Doppler showing umblical artery thrombosis	0 (.0)	2 (66.67)	0.002
**Past H/O IUGR**	No	29 (96.7)	1 (3.3)	0.042
	Yes	7 (70.0)	3 (30.0)	
**PastH/O Abortion early/late**	No	2 (100.0)	0 (.0)	1.000
	Yes	34 (89.5)	4 (10.5)	
**Past H/O DVT or PTE**	No	29 (87.9)	4 (12.1)	1.000
	Yes	7 (100.0)	0 (.0)	
**Drug history: Conception trial**	No	34 (89.5)	4 (10.5)	1.000
	Yes	2 (100.0)	0 (.0)	
**Ecosprin**	No	35 (89.7)	4 (18.2)	0.114
	Yes	1 (100.0)	0 (.0)	
**Heparin**	No	27 (100.0)	0 (.0)	0.008
	Yes	9 (69.2)	4 (30.8)	
**Multivitamins**	No	4 (80.0)	1 (20.0)	0.427
	Yes	32 (91.40)	3 (8.6)	
**Prochlorperazine**	No	36 (90.0)	4 (10.0)	1.000
	Yes	0 (.0)	0 (.0)	
**Progesterone support**	No	3 (100.0)	0 (.0)	1.000
	Yes	33 (89.2)	4 (10.8)	
**Folic acid**	No	32 (91.4)	3 (8.6)	0.427
	Yes	4 (80.0)	1 (20.0)	
**Amlong**	No	34 (94.4)	2 (5.6)	0.043
	Yes	2 (50.0)	2 (50.0)	
**Positive family history**	No	14 (100.0)	0 (.0)	1.000
	Yes	22 (84.6)	4 (15.4)	

We found that combined deficiency was more prevalent than isolated protein S deficiency. Protein S deficiency was more common in females during the first trimester, while combined deficiency was more prevalent in the second trimester. IUGR was reported in 70% of cases with combined deficiency only (Table 3).

**Table 3:** Comparative results of combined and isolated Protein C and S deficiency.

Combined protein C and S deficiency	Isolated protein S deficiency
Prevalence: 4/40	Prevalence: 2/40
All participants in second trimester	All participants in first trimester
Family history of abortion +ve for all	Family history of abortion +ve for all
No H/O stroke, DVT or PTE	No H/O stroke, DVT or PTE
3/4 had IUGR	No case of IUGR
Hypertension (n=2)	Hypertension (n=0)
All had past H/O abortions	All had past H/O abortions

All six participants with deficient protein C and S were treated with LMWH and were followed up until the end of pregnancy. They all delivered healthy babies, with APGAR scores of 10/10, and there were no reports of thromboembolic events.

## DISCUSSION

The activated proteins C and S, synthesized in the liver, play an important role in preventing blood coagulation *in vivo*. Protein C and S deficiency is not very common. This condition is particularly important in pregnant women as pregnancy is already a hypercoagulable state, and hypercoagulability caused by a deficiency of any antithrombotic factors leads to placental thrombosis, hypoperfusion, fetal death, and subsequent fetal losses. Our study aimed to compare the levels of protein C and protein S in pregnant females with repeated first and second-trimester pregnancy loss *versus* normal pregnant females [5, 6].

The mean age of females in the study group was 29.8±4.1 years, and in the control group, it was 29.4±4.3 years. A study by Sonal Vohra found that the median age of patients (n=381) with thrombophilia and unexplained pregnancy loss in Indian patients was 24 years (range: 16–41 years), while the median age of controls (n=100) was 24 years (range: 18–30 years), which is consistent with the age distribution in our study [7].

Regarding obstetric history, half of the women in the study group were gravida 4, followed by 10 who were G3, and 7 were G5. In the control group, 10 females were G2, 8 were G1 and G3 each, 7 were G5, and 5 were G4. In the study population, 37 females were para 0, and only 3 were para 1. In the control group, 13 were para 1, and 3 were para 2.

Twenty females in the study group were in their first and 20 in their second trimester. All 40 females were in the third trimester in the control group.

Fu *et al*. published the reference intervals for coagulation parameters in non-pregnant and pregnant women. Pregnant women with no significant history of miscarriages were distributed into 5 groups: 4–12, 13–20, 21–27, 28–33, and 34–42 weeks of gestation [8].

In our study, 33 cases had the ultrasound within normal limits, and 3 females had IUGR, of whom two showed reduced umbilical artery flow on Doppler, indicating suspected thrombosis. Similar results were observed by Samantha *et al*., who conducted a study on umbilical cord thrombosis: a meticulous culprit of intrauterine fetal demise. Twelve mothers (27.9%) consented to fetal autopsy in their study. The cause of death (COD) in all autopsies was umbilical cord thrombosis [9]. Similar findings were observed by Klaritsch *et al*., who studied spontaneous intrauterine umbilical artery thrombosis leading to IUGR, which was reported in G3 in the 31^st^ week of gestation. There were no signs of placental abruption or structural abnormalities except an absent paravesical color doppler flow in the region of the right umbilical artery, showing that umbilical artery thrombosis leads to fetal growth restriction [10].

3 females out of 40 had high blood pressure among cases with a mean systolic blood pressure (BP) of 119.4±11 and a mean diastolic BP of 74.2±8.3. Only 2 participants in the control group were hypertensive, with a mean systolic BP of 119.1±10.2 and diastolic BP of 73.8±7.7. These results agree with another study where 8 women out of 70 pregnant women had pregnancy-induced hypertension [11].

Regarding medical history, 38 females from the cases had a history of pregnancy loss, whereas none of the controls reported any history of pregnancy loss (p<0.001). Furthermore, 10 cases had a history of IUGR compared to 4 patients in the controls.

This data agrees with the study by Jyotsna *et al*., who worked on coagulation inhibitors and activated protein C resistance in recurrent pregnancy losses in Indian women [12].

Furthermore, 7 females in the study group had a history of deep venous thrombosis (DVT) and none among controls. Another study by Vora *et al*., who studied deep venous thrombosis during the antenatal period in Indian women, showed that 32 out of 34720 women had DVT during pregnancy, giving a prevalence of 0.1%. Seventeen women were in their first trimester, six in the second, and 9 in the third trimester [13].

Regarding treatment, 6 cases with recurrent pregnancy loss, hypertension, and a history of IUGR were put on Ecosprin, while 12 were prescribed LMWH, including women with reduced levels of protein C and S or H/O DVT. Thirty-seven cases were also prescribed progesterone. In a study by McNamee *et al*., 37 pregnant women were assessed for deficiencies in antithrombin, protein C, and protein S. Treatment for these deficiencies included the use of low molecular weight heparin and vitamin K antagonists. Out of the 26 women who received treatment, none experienced fetal losses. In contrast, 45% of the women who did not receive treatment (5 out of 11) experienced fetal loss [14].

Two randomized controlled trials compared aspirin with placebo or supportive care in women with antiphospholipid syndrome [15, 16]. It was observed that aspirin improves vasodilation and prostacyclin production and reduces the levels of thromboxane A2. It is commonly used to treat women with thrombophilia with recurrent miscarriages, who are also considered idiopathic.

Progesterone functions as an immunomodulator and shifts from a proinflammatory Th-1 cytokine response to an anti-inflammatory Th-2 cytokine response which is more pregnancy protective [17, 18]. However, other studies questioned the effectiveness of this treatment due to limitations in the methodology and inconsistent results of observational research [19].

Concerning the laboratory results, we found 9 cases with a positive ANA result compared to 1 participant in the control group. The presence of ANA in recurrent pregnancy loss indicates an underlying autoimmune disorder, which affects the development of the trophoblast leading to early pregnancy loss [20, 21].

In our study, protein C and S levels showed a significant drop in 4 out of 40 cases, but none of the controls had decreased levels of protein C or S. We also noticed a lower level of isolated free protein S in 2 cases.

Regarding the prevalence of protein C and S deficiency, our results are in accordance with a study by Mekaj *et al*. where the prevalence of AntiThrombin III, PC, and PS deficiencies was as follows: 2.88% (3/104), 3.85% (4/104), 5.77% (6/104), respectively. In the control group, the prevalence of ATIII deficiency was 0% (0/104), whereas a prevalence of 0.9% was found for both PC and PS deficiencies (1/110 each) [22].

Al Shammary *et al*. showed a significant correlation between low protein S with recurrent miscarriages [4]. In our study, 75% of cases with low protein C levels had IUGR on ultrasound, of whom 2 had reduced flow in the umbilical artery. They all had a positive family history of abortions and were treated with LMWH and progesterone support. In addition, 50% of patients with low protein S showed IUGR on ultrasound, of whom 2 had reduced flow in the umbilical artery. They all had past H/O abortions and were treated with LMWH and progesterone support. The common inherited thrombophilias, like protein C, protein S, and AT III, were significantly associated with unexplained pregnancy loss, and protein S deficiency had the strongest association [15].

Rey and his colleagues found that protein C and antithrombin deficiency were not associated with fetal loss, whereas protein S deficiency was linked with the late-term fetal loss [16]. The reason for infants being small for their gestational age remains unclear in most cases and may not be associated with inherited thrombophilia making it discordant with our study [23].

## CONCLUSION

Our study showed that combined deficiency of protein C and S was present in 10% of cases, and isolated protein S deficiency was present in 5% of cases, compared to zero results in controls. These results suggest that protein C and S deficiency may be one of the causative factors for recurrent pregnancy loss, although our sample size was limited to draw a clear inference between recurrent pregnancy loss and protein C and S deficiency.

Our study will pave the way for more such research to be conducted in the near future so that we can better understand the prevalence and significance of protein C and S deficiency in recurrent pregnancy loss. All these 6 cases were treated with low molecular weight heparin and progesterone support, and they all had healthy offspring upon follow-up. Therefore, screening for protein C and S deficiency is mandatory in all cases of recurrent pregnancy loss, and treatment with LMWH and progesterone should be initiated to ensure good fetal outcomes and prevent post-partum/postoperative venous thromboembolism.

## Data Availability

Further data is available from the corresponding author on reasonable request.

## References

[1] Esmon C, Vigano D, Angelo S, Dangelo A, Comp P (1987). Anticoagulation proteins C and S. Advanced Exp Med Biol.

[2] Alshammary H, Almosawi H, Hadi F (2015). Deficiency of Protein C and Protein S in Recurrent Pregnancy Loss. Medical Journal of Babylon.

[3] Rosenberg R, Rosenberg J (1984). Natural anticoagulant mechanisms. Journal of Clinnical Investigation.

[4] Lalan DM, Jassawalla MJ, Bhalerao SA (2012). Successful pregnancy outcome in a case of protein s deficiency. J Obstet Gynaecol India.

[5] Chaudhari H, Shah P, Pai K, DSouza N (2016). Combined protein C and protein S deficiency with pregnancy. International Journal of Reprod Contracept Obstet and Gynaecol.

[6] Warwick R, Hutton R, Goff L, Letsky E, Heard M (1989). Changes in protein C and free protein S during pregnancy and following hysterectomy. Journal of Royal Society of Medicine.

[7] Vora S, Shetty S, Salvi V, Satoskar P, Ghosh K (2008). Thrombophilia and unexplained pregnancy loss in Indian patients. Natl Med J India.

[8] Fu M, Liu J, Xing J, Dai Y (2022). Reference intervals for coagulation parameters in non-pregnant and pregnant women. Scientific Reports.

[9] Stancu S, Poalelungi C, Carabas B, Rusu M (2016). Umbilical cord thrombosis: a meticulous culprit of intrauterine fetal demise. EJPMR.

[10] Klaritsch P, Haeusler M, Karpf E, Schlembach D, Lang U (2008). Spontaneous intrauterine umbilical artery thrombosis leading to severe fetal growth restriction. Placenta.

[11] Franklin DK (2004). Hypertensive Disorders of Pregnancy: Prevalence, Maternal Complications and Perinatal Outcomes at Lilongwe Central Hospital, Malawi. Department of General Practice and Community Medicine Faculty of Medicine, University of Oslo, Norway.

[12] Jyotsna PL, Sharma S, Trivedi SS (2011). Coagulation inhibitors and activated protein C resistance in recurrent pregnancy losses in Indian women. Indian J Pathol Microbiol.

[13] Vora S, Ghosh K, Shetty S, Salvi V, Satoskar P (2007). Deep venous thrombosis in the antenatal period in a large cohort of pregnancies from western India. Thromb J.

[14] McNamee K, Dawood F, Farquharson RG (2012). Thrombophilia and early pregnancy loss. Best Pract Res Clin Obstet Gynaecol.

[15] Tulppala M, Marttunen M, Söderstrom-Anttila V, Foudila T (1997). Low-dose aspirin in prevention of miscarriage in women with unexplained or autoimmune related recurrent miscarriage: effect on prostacyclin and thromboxane A2 production. Hum Reprod.

[16] Pattison NS, Chamley LW, Birdsall M, Zanderigo AM (2000). Does aspirin have a role in improving pregnancy outcome for women with the antiphospholipid syndrome? A randomized controlled trial. Am J Obstet Gynecol.

[17] Hirahara F, Andoh N, Sawai K, Hirabuki T (1998). Hyperprolactinemic recurrent miscarriage and results of randomized bromocriptine treatment trials. Fertil Steril.

[18] Raghupathy R, Al Mutawa E, Makhseed M, Azizieh F, Szekeres-Bartho J (2005). Modulation of cytokine production by dydrogesterone in lymphocytes from women with recurrent miscarriage. BJOG.

[19] Kaandorp S, Di Nisio M, Goddijn M, Middeldorp S (2009). Aspirin or anticoagulants for treating recurrent miscarriage in women without antiphospholipid syndrome. Cochrane Database Syst Rev.

[20] Mavragani CP, Ioannidis JP, Tzioufas AG, Hantoumi IE, Moutsopoulos HM (1999). Recurrent Pregnancy Loss and Autoantibody Profile in Autoimmune Diseases. Rheumatol (Ox).

[21] Ticconi C, Pietropolli A, Borelli B, Bruno V (2016). Antinuclear Autoantibodies and Pregnancy Outcome in Women With Unexplained Recurrent Miscarriage. Am J Reprod Immunol.

[22] Mekaj Y,Lulaj S, Daci F, Rafuna N (2015). Prevalence and role of antithrombin III, protein C and protein S deficiencies and activated protein C resistance in Kosovo women with recurrent pregnancy loss during first trimester of pregnancy. J Hum Reprod Sci.

[23] Lindhoff-Last E (2004). Maternal thrombophilia and obstetric complications. J Lab Med.

